# Supplementary figures and images for: Wolfram syndrome 1 regulates sleep in dopamine receptor neurons by modulating calcium homeostasis
Source: PLoS Genet. 2023 Jul 3;19(7):e1010827. doi: 10.1371/journal.pgen.1010827 (PMC10348591; doi:10.1371/journal.pgen.1010827)

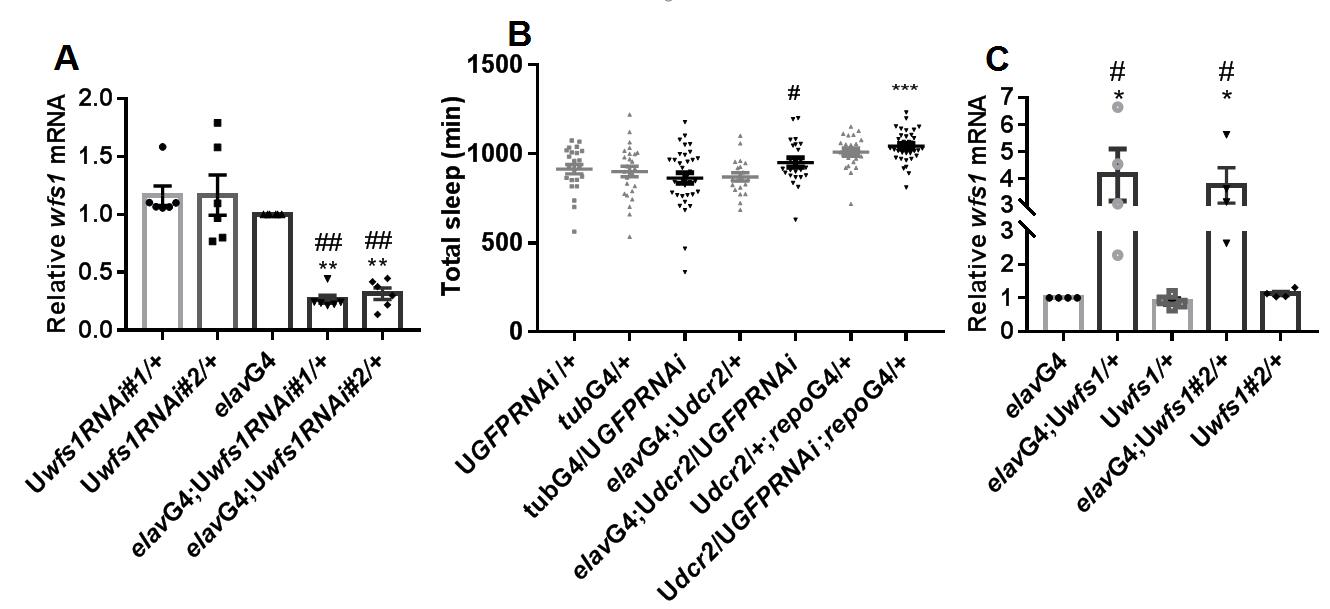

Supplement: S1 Fig — (A) and (C) Relative mRNA abundance of wfs1 determined by qRT–PCR in whole-head extracts. (B) Daily sleep duration of GFP RNAi flies. n = 20–35. For comparison between RNAi flies vs. UAS/GAL4 controls, one-way ANOVA was used: compared to GAL4 control, #P < 0.05; compared to UAS control, ***P < 0.001. For (A) and (C), the value of GAL4 control at was set to 1. n = 4–6. Error bars represent SEM. Mann-Whitney test: compared to GAL4 control, #P < 0.05, ##P < 0.01; compared to UAS control, *P < 0.05, **P < 0.01. G4, GAL4; U, UAS. (TIF) [file pgen.1010827.s001.tif]

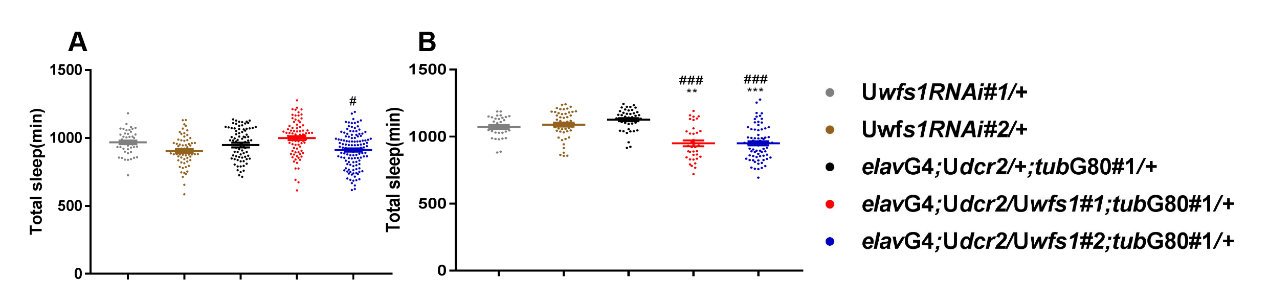

Supplement: S2 Fig — Daily sleep duration of flies with wfs1 knocked down in adult stage (A) or developmental (B) stage. n = 31–126. For comparison between RNAi flies vs. UAS/GAL4 controls, one-way ANOVA was used: compared to GAL4 control, #P < 0.05, ###P < 0.001; compared to UAS control, **P < 0.01, ***P < 0.001. G4, GAL4; U, UAS; G80, GAL80. (TIF) [file pgen.1010827.s002.tif]

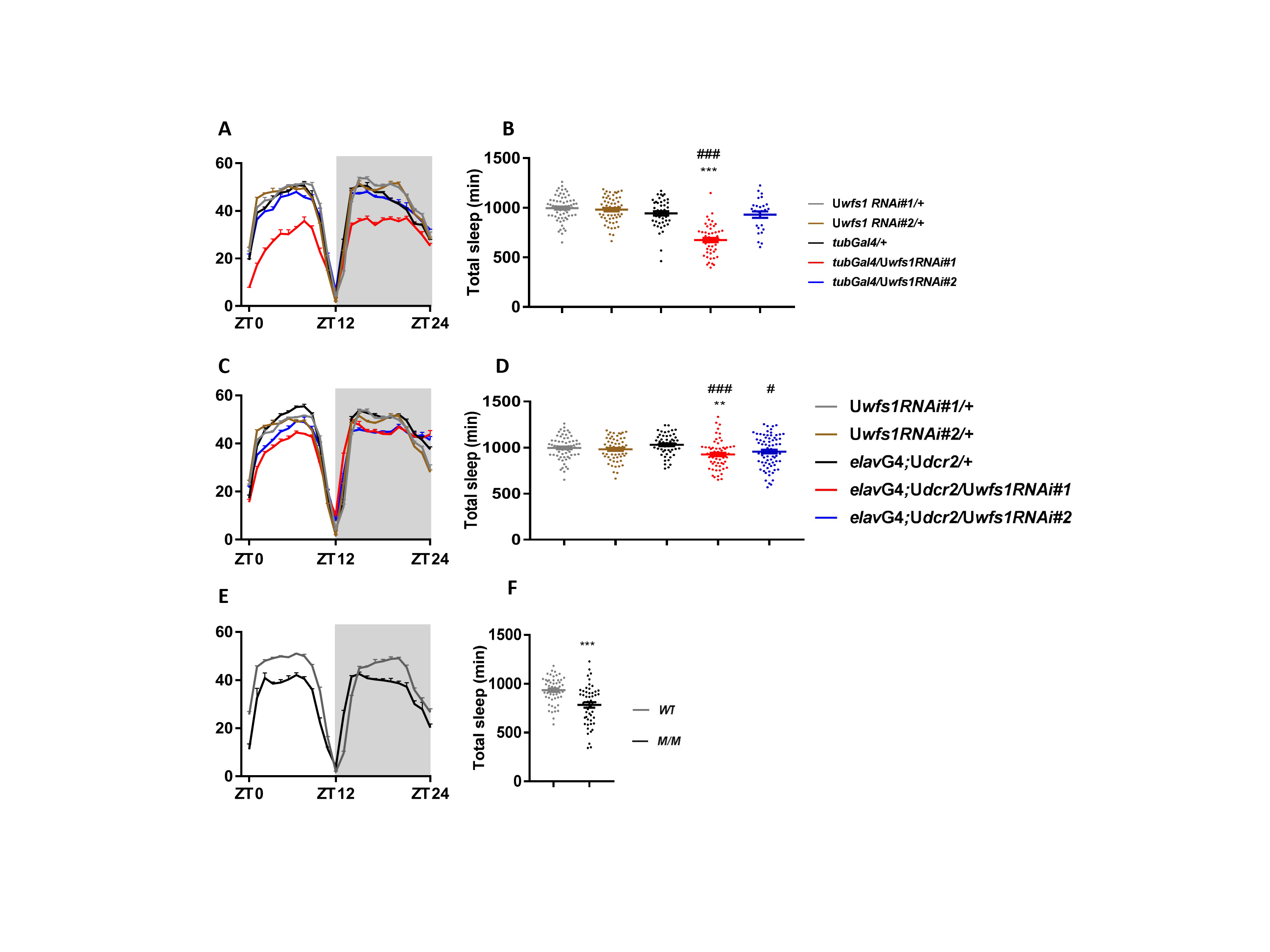

Supplement: S3 Fig — (A, C and E) The sleep profile of wfs1 RNAi flies, wfs1 mutants and controls collected 30 days post-eclosion. (B, D and F) The sleep profile of flies, wfs1 mutants and controls collected 30 days post-eclosion. Gray shade indicates the dark period. For comparison between RNAi flies vs. UAS/GAL4 controls, one-way ANOVA was used: compared to GAL4 control, #P < 0.05, ###P < 0.001; compared to UAS control, **P < 0.01, ***P < 0.001. For comparison between mutant vs. control, Mann-Whitney test or Student’s t-test was used: ***P < 0.001. n = 26–73. Error bars represent SEM. G4, GAL4; U, UAS; M, wfs1MI14041; ZT, Zeitgeber Time. (TIF) [file pgen.1010827.s003.tif]

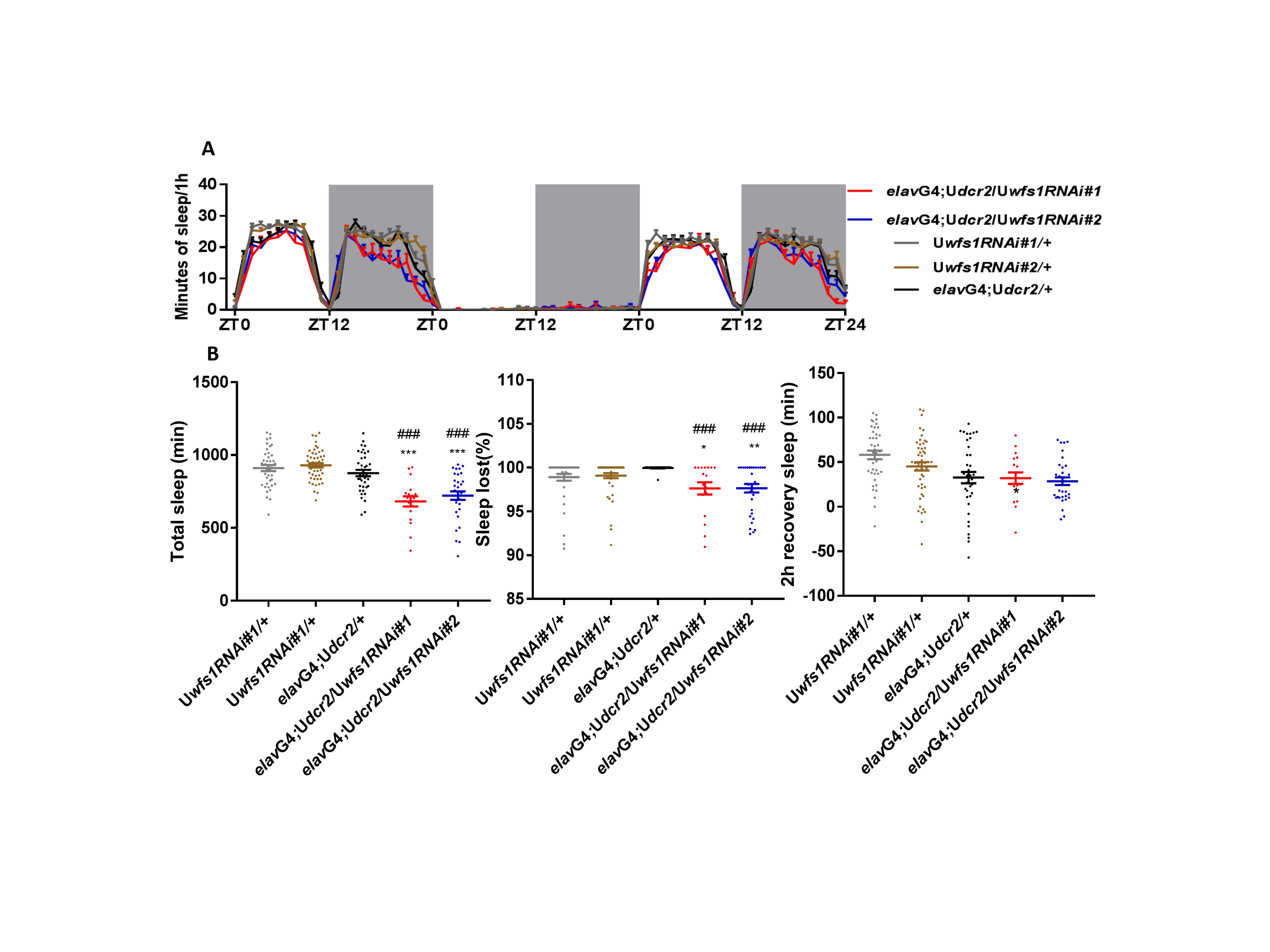

Supplement: S4 Fig — (A) Sleep profile of wfs1 RNAi and control flies the day before, during and after sleep mechanical deprivation. White and black bars indicate light and dark period, respectively. (B) Left panel: sleep duration the day before sleep deprivation. Middle panel: percentage of sleep lost on the day of sleep deprivation. Right panel: The recovery sleep during the first 2 h on the day immediately after sleep deprivation. One-way ANOVA: compared to GAL4 control, ###P < 0.001; compared to UAS control, *P < 0.05, **P < 0.01, ***P < 0.001. n = 18–49. Error bars represent SEM. G4, GAL4; U, UAS; ZT, Zeitgeber Time. (TIF) [file pgen.1010827.s004.tif]

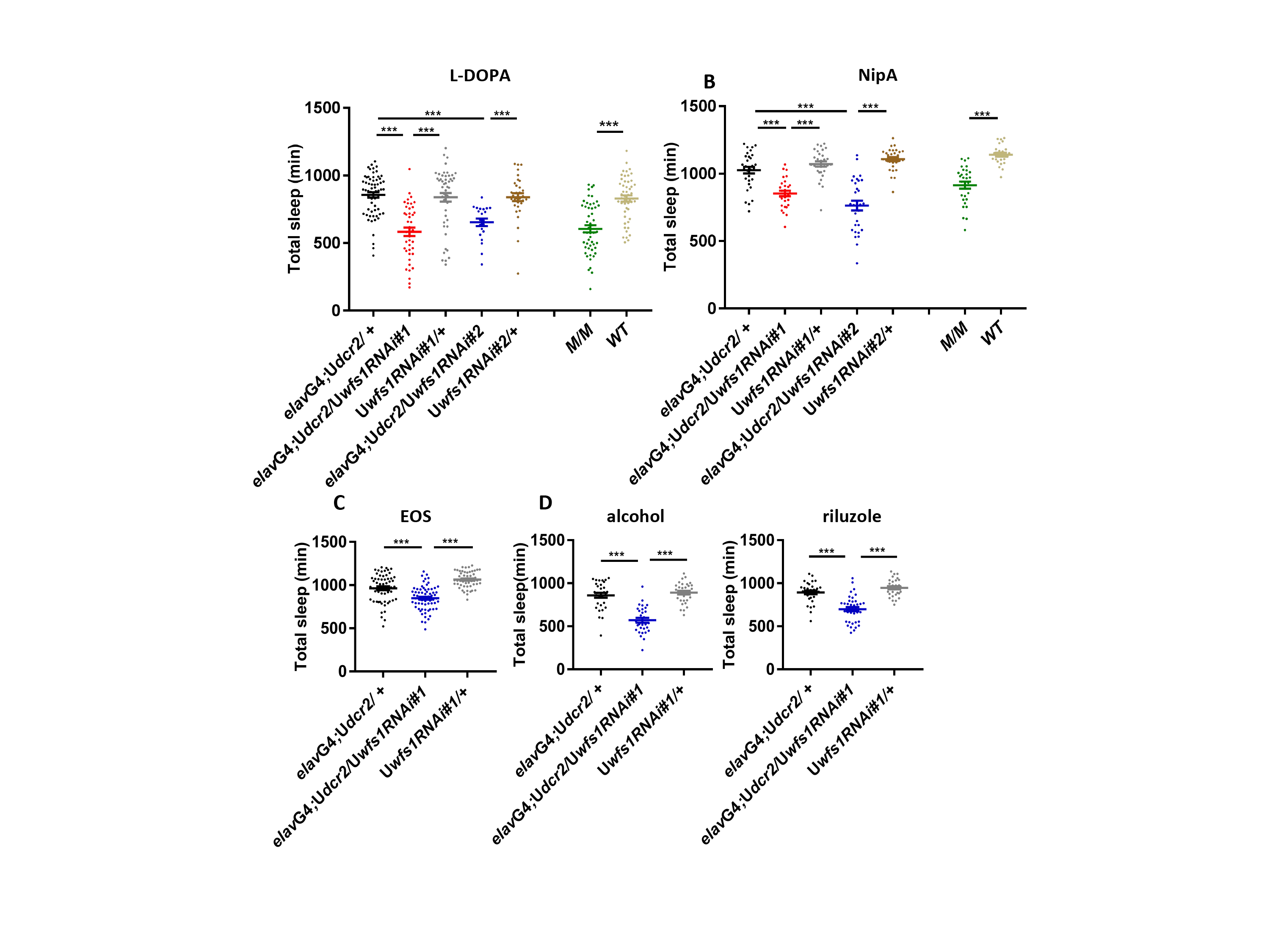

Supplement: S5 Fig — Daily sleep duration of wfs1 RNAi, mutant and control flies treated with the indicated drugs. Alcohol is used as a vehicle control for riluzole, while all other drugs were directly dissolved in the food. For comparison between RNAi flies vs. UAS/GAL4 controls, one-way ANOVA was used. For comparison between mutants expressing RNAi vs. mutant control, Student’s t-test was used. ***P < 0.001. n = 21–67. Error bars represent SEM. G4, GAL4; U, UAS; M, wfs1MI14041; NipA, nipecotic acid; EOS, ethanolamine-O-sulphate. (TIF) [file pgen.1010827.s005.tif]

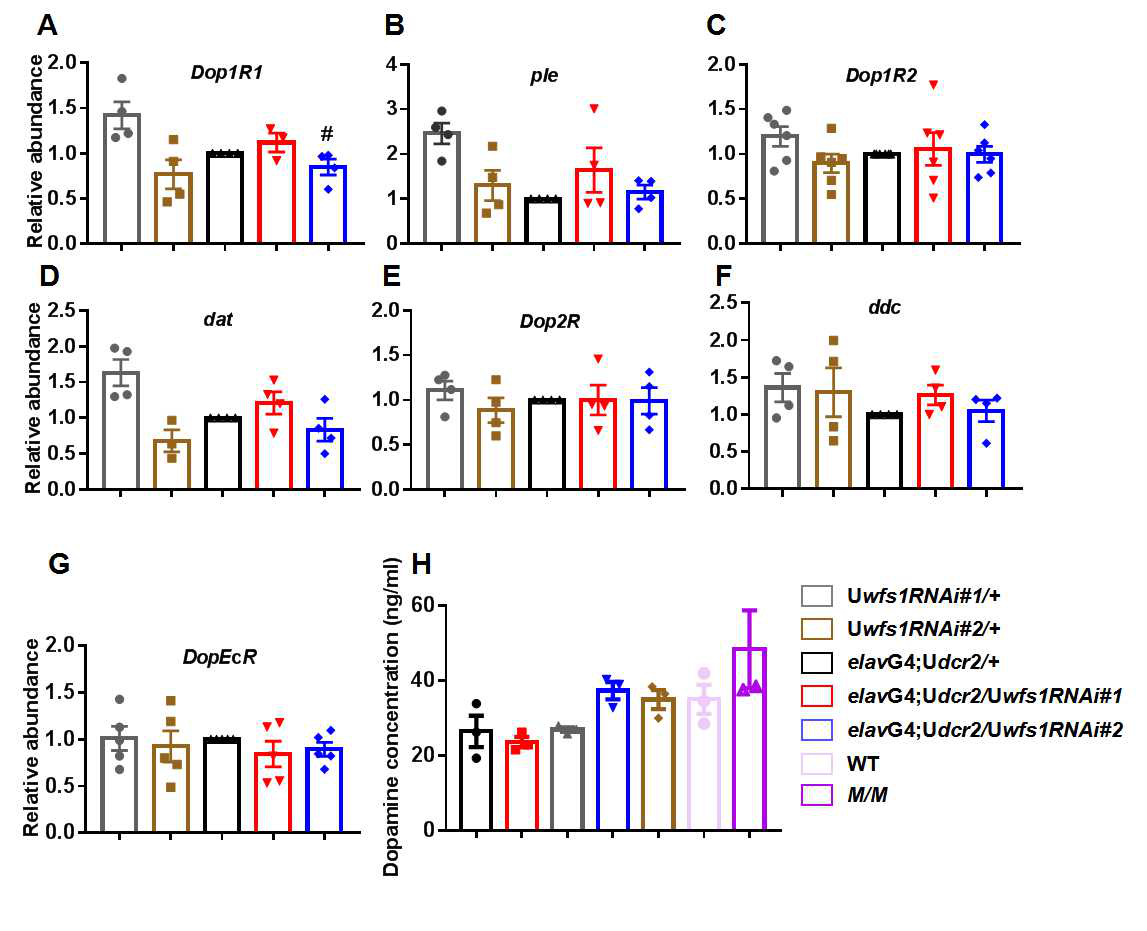

Supplement: S6 Fig — (A-G) Relative mRNA abundance of genes involved in dopamine signaling in whole-head extracts, determined by qRT-PCR. For each experiment, the value of G4 control was set to 1. (H) Dopamine concentration in whole head extracts determined by ELISA. Error bars represent SEM. n = 3–6. Mann-Whitney test: compared to GAL4 control, #P < 0.05. G4, GAL4; U, UAS; M, wfs1MI14041. (TIF) [file pgen.1010827.s006.tif]

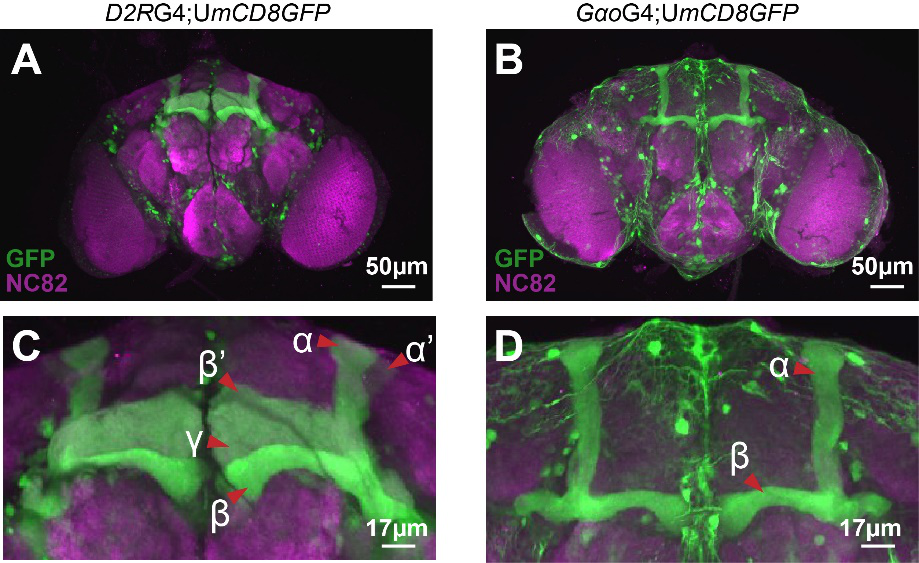

Supplement: S7 Fig — (A and B) Representative image of adult fly brain with mCD8GFP expression in Dop2R (A) or Goα+ (B) cells. (C) and (D) are enlarged image of MB shown in (A) and (C), respectively. The brains are stained with antibody against BRUCHPILOT (NC82) to label axons. Red arrowhead indicates the MB. The scale bar represents 50 μm and 17 μm, respectively. D2RG4, Dop2RGAL4; G4, GAL4; U, UAS. (TIF) [file pgen.1010827.s007.tif]

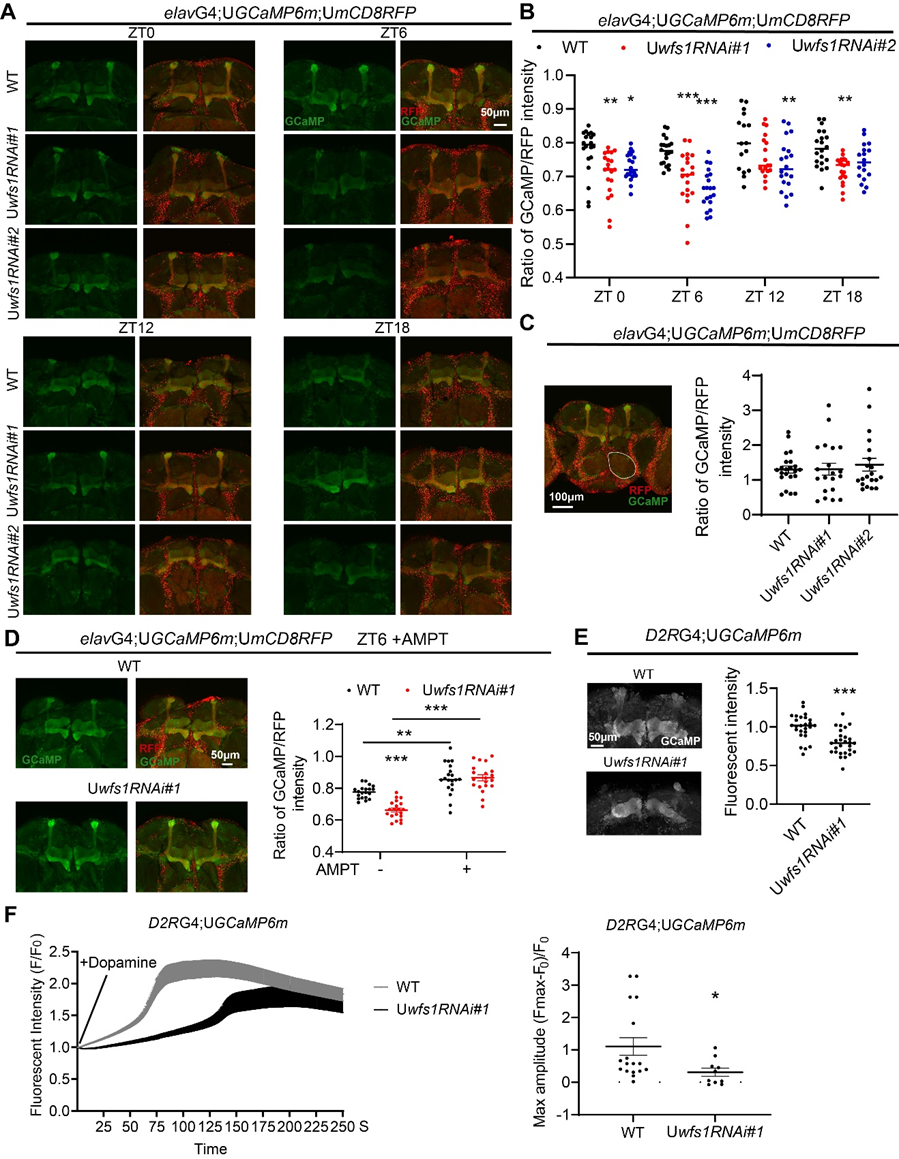

Supplement: S8 Fig — Representative live image of adult fly brain with pan-neuronal expression of GCaMP6m and mCD8RFP. Brain samples were collected and dissected at the indicated time points. (B) Quantification of GCaMP6m/RFP signal intensity in MB (two-way ANOVA, n = 16–20). (C) Left panel: representative live image of adult fly brain with pan-neuronal expression of GCaMP6m and mCD8RFP with the antennal lobe indicated by the white rectangle. Brain samples were collected and dissected at ZT6. Right panel: quantification of antennal lobe GCaMP6m/RFP intensity (one-way ANOVA, n = 22, 20, 22). (D) Left panel: representative live image of adult fly brain with pan-neuronal expression of GCaMP6m and mCD8RFP. The flies were fed with 2 mM AMPT for 3 days. Brain samples were collected and dissected at ZT6. Right panel: quantification of GCaMP6m/RFP signal intensity in MB (two-way ANOVA, n = 20). (E) Left panel: representative live image of adult fly brain with GCaMP6m expressed in Dop2R neurons. Brain samples were collected and dissected at ZT6. Right panel: quantification of MB GCaMP6m signal intensity normalized to the control (Student’s t-test, n = 26, 28). (F) Left panel: time-series GCaMP6m intensity of adult fly brain treated with dopamine. GCaMP6m intensity was normalized to the baseline level. Right panel: quantification of maximum GCaMP6m intensity after dopamine treatment (Student’s t-test, n = 17, 10). Error bars represent SEM. *P < 0.05, **P < 0.01, ***P < 0.001. The scale bar represents 50 μm unless indicated otherwise. G4, GAL4; U, UAS; D2RGAL4, Dop2RGAL4. ZT, Zeitgeber Time. (TIF) [file pgen.1010827.s008.tif]

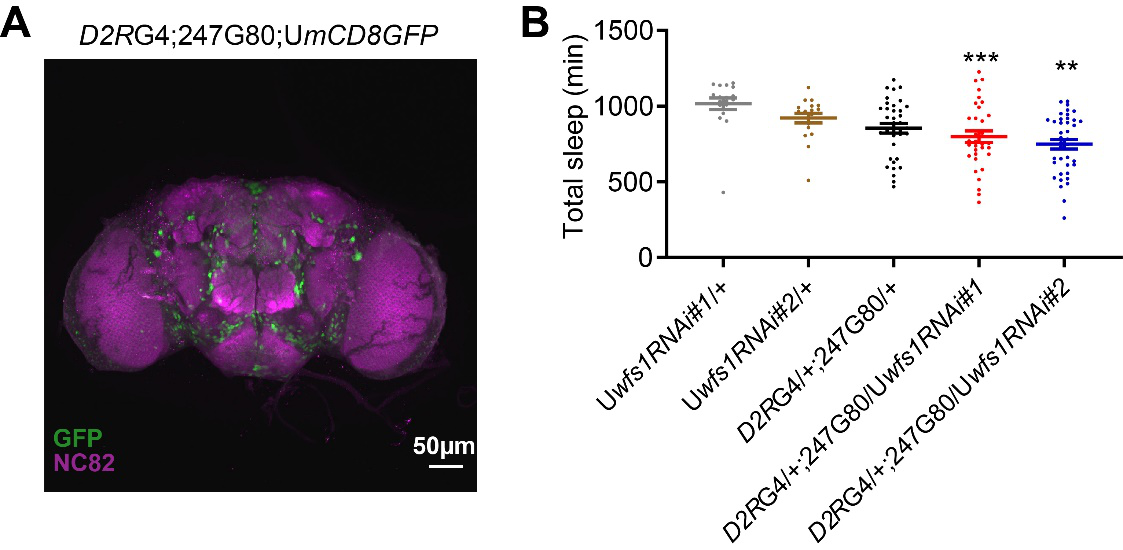

Supplement: S9 Fig — (A) Representative image of adult fly brain with mCD8GFP expression in non-MB Dop2R neurons. The brains are stained with antibody against BRUCHPILOT (NC82) to label axons. The scale bar represents 50 μm. (B) Daily sleep duration of wfs1RNAi flies and controls. For comparison between RNAi flies vs. UAS/GAL4 controls, one-way ANOVA was used: compared to UAS control, **P < 0.01, ***P < 0.001. n = 18–39. Error bars represent SEM. D2RG4, Dop2RGAL4; U, UAS; G80, GAL80. (TIF) [file pgen.1010827.s009.tif]

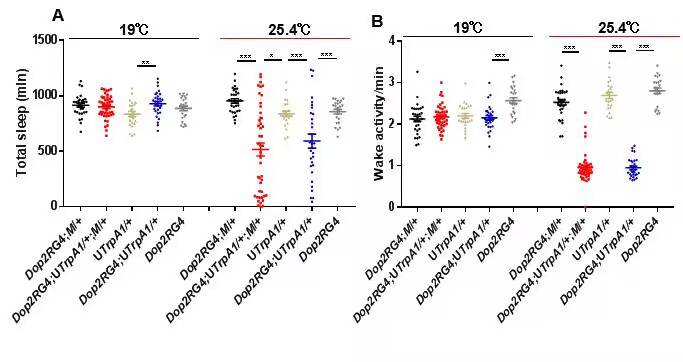

Supplement: S10 Fig — (A and B) Daily sleep duration (A) and wake activity (B) of wfs1 heterozygous mutant flies with Dop2R neurons activated by TrpA1. For comparison between RNAi flies vs. UAS/GAL4 controls, one-way ANOVA was used: **P < 0.01, ***P < 0.001. For comparison between mutants expressing TrpA1 vs. mutant control, Student’s t-test was used: ***P < 0.001. n = 24–45. Error bars represent SEM. D2RG4, Dop2RGAL4; U, UAS; M, wfs1MI1404. (TIF) [file pgen.1010827.s010.tif]

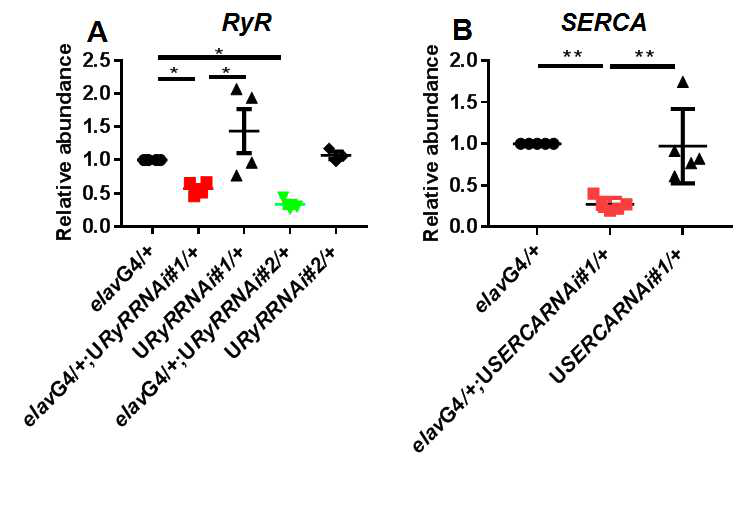

Supplement: S11 Fig — (A and B) Relative mRNA abundance of RyR (A) and SERCA (B) determined by qRT-PCR in whole-head extracts. Female fly heads were used for this entire experiment as we were not able to obtain male elavGAL4;URyRRNAi#1/+ flies. We were not able to obtain either male or female elavGAL4;USERCARNAi#2/+ flies. For each experiment, the value of G4 control at was set to 1. Error bars represent SEM. n = 3–5. Mann-Whitney test: *P < 0.05, **P < 0.01. G4, GAL4; U, UAS. (TIF) [file pgen.1010827.s011.tif]

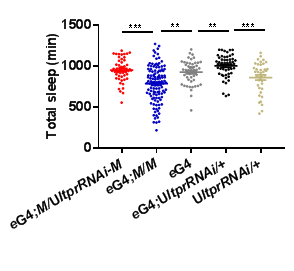

Supplement: S12 Fig — Daily sleep duration of wfs1 mutant flies with Itpr knocked down in all neurons. For comparison between RNAi flies vs. UAS/GAL4 controls, one-way ANOVA was used. For comparison between mutants expressing RNAi vs. mutant control, Student’s t-test was used: **P < 0.01, ***P < 0.001. n = 38–116. Error bars represent SEM. eG4, elavGAL4; U, UAS; M, wfs1MI14041. (TIF) [file pgen.1010827.s012.tif]
