# Supplementary material for: Wolfram syndrome 1 regulates sleep in dopamine receptor neurons by modulating calcium homeostasis
Source: PLoS Genet. 2023 Jul 3;19(7):e1010827. doi: 10.1371/journal.pgen.1010827 (PMC10348591; doi:10.1371/journal.pgen.1010827)
Supplement: S1 Table — (DOCX) [file pgen.1010827.s016.docx]

**S1 Table. Locomotor rhythm of flies with *wfs1* knocked down during adult or development.**

| **Genotype** | **Period±SEM (hour)** | **Power±SEM** | **Rhythmicity** | **N** |
| --- | --- | --- | --- | --- |
| **Uwfs1RNAi#1/+;tubG80^ts^#1/+ (18-29℃)** | 23.14±0.3 | 74.27±7.54 | 88% | 42 |
| **Uwfs1RNAi#2/+;tubG80^ts^#1/+ (18-29℃)** | 22.95±0.04 | 44.25±4.81 | 94% | 34 |
| ***elav*G4;Udcr2/+ (18-29℃)** | 23.04±0.06 | 64.03±7.57 | 89% | 27 |
| ***elav*G4;Udcr2/Uwfs1RNAi#1; tubG80ts#1/+ (18-29****℃)** | 22.93±0.03 | 59.9±5.22 | 90% | 51 |
| ***elav*G4;Udcr2/Uwfs1RNAi#2; tubG80ts#1/+ (18-29****℃)** | 22.93±0.04 | 78.72±5.95 | 98% | 46 |
| **Uwfs1RNAi#1/+;tubG80^ts^#1/+ (29-18℃)** | 23.54±0.2 | 26.62±5.22 | 52% | 27 |
| **Uwfs1RNAi#2/+;tubG80^ts^#1/+ (29-18℃)** | 23.78±0.13 | 30.54±6.16 | 78% | 23 |
| ***elav*G4;Udcr2/+ (29-18℃)** | 23.86±0.21 | 6.36±1.54 | 25% | 28 |
| ***elav*G4;Udcr2/Uwfs1RNAi#1; tubG80ts#1/+ (29-18℃)** | 23.9±0.29 | 8.62±2.95** | 23% | 22 |
| ***elav*G4;Udcr2/Uwfs1RNAi#2; tubG80ts#1/+ (29-18℃)** | 23.8±0.2 | 6.18±2.93*** | 17% | 30 |

For comparison between RNAi flies vs. UAS/GAL4 controls, one-way ANOVA was used: compared to GAL4 control, ##*P* < 0.01, ###*P* < 0.001; compared to UAS control, ***P* < 0.01, ****P* < 0.001. G4, GAL4; U, UAS; G80, GAL80.
