# Supplementary material for: Wolfram syndrome 1 regulates sleep in dopamine receptor neurons by modulating calcium homeostasis
Source: PLoS Genet. 2023 Jul 3;19(7):e1010827. doi: 10.1371/journal.pgen.1010827 (PMC10348591; doi:10.1371/journal.pgen.1010827)
Supplement: S2 Table — (DOCX) [file pgen.1010827.s017.docx]

**S2 Table. *Wfs1* deficiency dampens locomotor rhythm in aged flies.**

| **Genotype** | **Period±SEM (hour)** | **Power±SEM** | **Rhythmicity** | **N** |
| --- | --- | --- | --- | --- |
| **U*wfs1RNAi#1/+*** | 23.99±0.05 | 38.42±2.81 | 87% | 68 |
| **U*wfs1RNAi#2/+*** | 23.94±0.05 | 41.56±2.73 | 94% | 67 |
| ***tub*G4*/+*** | 24.28±0.05 | 36.45±3.42 | 79% | 61 |
| ***tub*G4*/*U*wfs1RNAi#1*** | 24.62±0.25** | 10.47±2.14***### | 38% | 56 |
| ***tub*G4*/*U*wfs1RNAi#2*** | 24±0 | 12.14±11.39**# | 13% | 8 |
| ***elav*G4*;*U*dcr2/+*** | 24.01±0.06 | 27.75±3.59 | 70% | 53 |
| ***elav*G4*;*U*dcr2/*U*wfs1RNAi#1*** | 24.35±0.16 | 5.62±1.26***### | 17% | 58 |
| ***elav*G4*;*U*dcr2/*U*wfs1RNAi#2*** | 24.33±0.16 | 6.86±1.66***### | 21% | 42 |
| **WT** | 24.23±0.13 | 32.15±2.93 | 89% | 45 |
| ***M/M*** | 23.67±0.2& | 11.76±2.58@@@ | 36% | 33 |

For comparison between RNAi flies vs. UAS/GAL4 controls, one-way ANOVA was used: compared to GAL4 control, ##*P* < 0.01, ###*P* < 0.001; compared to UAS control, ***P* < 0.01, ****P* < 0.001. For comparison between mutant vs. control, Mann-Whitney test was used: compared to WT, @@@*P* < 0.001; G4, GAL4; U, UAS; *M*, *wfs1^MI14041^*.
