# Supplementary material for: Wolfram syndrome 1 regulates sleep in dopamine receptor neurons by modulating calcium homeostasis
Source: PLoS Genet. 2023 Jul 3;19(7):e1010827. doi: 10.1371/journal.pgen.1010827 (PMC10348591; doi:10.1371/journal.pgen.1010827)
Supplement: S3 Table — (DOCX) [file pgen.1010827.s018.docx]

**S3 Table. The locomotor rhythm of *wfs1* mutant flies with *ple* or *Dop2R* knocked down.**

| **Genotype** | **Period±SEM (hour)** | **Power±SEM** | **Rhythmicity** | **N** |
| --- | --- | --- | --- | --- |
| ***elav*G4;*M*/U*pleRNAi*-*M*** | 23.56±0.07 | 54.23±7.19$$$ | 75% | 44 |
| ***elav*G4;*M/M*** | 23.67±0.11 | 10.99±3.42@@@ | 21% | 42 |
| ***elav*G4** | 23.96±0.03 | 64.71±4.97 | 91% | 67 |
| ***elav*G4;U*pleRNAi*/+** | 23.84±0.03 | 89.52±6.02***## | 98% | 46 |
| **U*pleRNAi*/+** | 23.76±0.05 | 51.87±5.61 | 82% | 44 |
| ***elav*G4*;*U*Dop2RRNAi/+;M/M*** | 23.93±0.13 | 19.83±8.46 | 32% | 22 |
| ***elav*G4*;*U*Dop2RRNAi/+*** | 23.74±0.05 | 98.3±6.06**### | 97% | 36 |
| **U*Dop2RRNAi/+*** | 23.78±0.06 | 58.08±8.35 | 85% | 27 |

For comparison between RNAi flies vs. UAS/GAL4 controls, one-way ANOVA was used: compared to GAL4 control, ##*P* < 0.01, ###*P* < 0.001; compared to UAS control, ***P* < 0.01, ****P* < 0.001. For comparison between mutant vs. control, Mann-Whitney test was used: compared to WT, @@@*P* < 0.001; For comparison between mutant expressing RNAi vs. mutant control, one-way ANOVA was used: $$$*P* < 0.001. G4, GAL4; U, UAS; *M*, *wfs1^MI14041^*.
