# Supplementary material for: Wolfram syndrome 1 regulates sleep in dopamine receptor neurons by modulating calcium homeostasis
Source: PLoS Genet. 2023 Jul 3;19(7):e1010827. doi: 10.1371/journal.pgen.1010827 (PMC10348591; doi:10.1371/journal.pgen.1010827)
Supplement: S4 Table — (DOCX) [file pgen.1010827.s019.docx]

**S4 Table. Oligonucleotides used in this study.**

| **Genes** | **Sense (5’-3’)** | **Antisense (5’-3’)** | **Source** |
| --- | --- | --- | --- |
| ***wfs1*** | CTTCTGGTGCCTGTCTTCGT | CAACAAGCACGCTGTTGTGA | [1] |
| ***dat*** | CAGATGTCACCAACCGGACA | GAACAGAGGAATGCCACCGA | This paper |
| ***ddc*** | CGCCGGAGTTCAAGGATTTT | GCATCCGGAATCAATGGCTT | This paper |
| ***Dop1R1*** | GCGTGGTCATGATTGGCATC | ATGCTCTTGTACCGCTGCTT | This paper |
| ***ple*** | CATCTTCCAGAGCACCCAGT | ATGTTCCTTGCAGAGACCGA | This paper |
| ***DopEcR*** | ACCTCCTCATTATCGCCACC | TCGCCGTACATCCATTCTCC | This paper |
| ***Dop1R2*** | GTCTGGGTTCTGCATCGAGT | CAAAGGCCCTGCGAAAGTC | This paper |
| ***Dop2R*** | GAGCGCAAGGCCACCAAAACA | GGGTTCACAAAGCTGTTGATGTAG | This paper |
| ***per*** | CAGCAGCAGCCTAATCG | GAGTCGGACACCTTGG | [2] |
| ***clk*** | TACTGCGTGAGGATATCG | GTTGTTGTTCTGGTTGC | [2] |
| ***tim*** | TTCTCCTCCTTGGGTTGCTT | ATTCTCCAGCAGCGGTATCA | This paper |
| ***cry*** | TGCAGGTACCAAGAATGTGG | GGAAGATAAGCCGGTTCG | [3] |
| ***RyR*** | CGACCTTATCGAATCGCCCA | CGGTTCGTTTGGCATGTGTT | This paper |
| ***SERCA*** | AGGCTAACCAGAAGAAATACGGA | GGCGAGAACAAATGAGATGATGG | This paper |
| ***rp49*** | TACAGGCCCAAGATCGTGAA | GCACTCTGTTGTCGATACCC | [2] |

**References**

1. Sakakibara Y, Sekiya M, Fujisaki N, Quan X, Iijima KM. Knockdown of wfs1, a fly homolog of Wolfram syndrome 1, in the nervous system increases susceptibility to age- and stress-induced neuronal dysfunction and degeneration in Drosophila. PLoS Genet. 2018;14(1):e1007196. Epub 2018/01/23. doi: 10.1371/journal.pgen.1007196. PubMed PMID: 29357349; PubMed Central PMCID: PMCPMC5794194.

2. Bu B, He W, Song L, Zhang L. Nuclear Envelope Protein MAN1 Regulates the Drosophila Circadian Clock via Period. Neuroscience bulletin. 2019;35(6):969-78. doi: 10.1007/s12264-019-00404-6. PubMed PMID: 31230212; PubMed Central PMCID: PMCPMC6864020.

3. Bu B, Chen L, Zheng L, He W, Zhang L. Nipped-A regulates the Drosophila circadian clock via histone deubiquitination. EMBO J. 2020;39(1):e101259. Epub 2019/09/21. doi: 10.15252/embj.2018101259. PubMed PMID: 31538360; PubMed Central PMCID: PMCPMC6939192.
