# Supplementary material for: Wolfram syndrome 1 regulates sleep in dopamine receptor neurons by modulating calcium homeostasis
Source: PLoS Genet. 2023 Jul 3;19(7):e1010827. doi: 10.1371/journal.pgen.1010827 (PMC10348591; doi:10.1371/journal.pgen.1010827)
Supplement: Attachment — Submitted filename: response to reviewers.docx [file pgen.1010827.s020.docx]

***We thank the reviewers for their thoughtful comments and suggestions which have made the manuscript much stronger. We have addressed these comments and suggestions as described below. The original reviews are listed point-by-point. Our responses are in blue italic font.* *Edits made in the text of the manuscript are marked in red.***

Reviewer #1: The authors found that in Drosophila, the inhibition of wfs1 causes increased arousal, resulting in reduced sleep. This phenotype was shown to involve dopamine signaling along with ER Ca2+ dynamics. Altered ER Ca2+ dynamics in wfs1 mutations has already been reported, but this manuscript may be the first report to show a sleep behavior in flies. Each experiment was carefully analyzed. However, there are some problems with the in vivo experiments, which are listed below.

The biggest problem is the Ca2+ imaging experiment in Fig. 5. These are all observed at one time point after dissection to estimate basal Ca2+ concentration and Ca2+ concentration during neural activity. These methods are indirect and it is questionable whether they accurately measure neural activity-dependent Ca2+ dynamics. Ca2+ dynamics before and after dopamine (DA) stimulation should be shown in the same samples.

*Thank you for this important suggestion. We have now examined the effect of knocking down wfs1 on dopamine-induced changes of calcium level using GCaMP6. We found that dopamine elicits a significantly smaller calcium increase in Dop2R neurons of wfs1 RNAi flies (Fig S8F). However, we have conducted further literature search and found that wolframin has been shown to bind to calmodulin (CaM) in rat brain, and is capable of binding with calcium/CaM complex in vitro and in transfected cells (Yurimoto et al., 2009). This may undermine the validity of using GCaMP to monitor calcium level in wfs1 deficient animals and cells, and could potentially account for the contradictory data acquired using CaLexA vs GCaMP. Therefore, we believe that CaLexA and spH are more likely to reflect the true changes of neural activity in wfs1 deficient flies, which both point to hyperexcitability. This elevated activity is consistent with our behavioral data, as activation of Dop2R/Goα+ cells reduces sleep, similar to the effects of wfs1 deficiency. Moreover, silencing Dop2R neurons rescues the short-sleep phenotype of wfs1 mutants, while over-expressing wfs1 restores the decreased sleep induced by activation of Dop2R neurons (Fig 6D and 6E). Taken together, these findings indicate that wolframin functions to suppress the excitability of Dop2R neurons, which in turn reduces wakefulness and promotes sleep.*

The authors seem to focus on Ca2+ alteration in the mushroom body (MB). However, it is possible that ER Ca2+ alteration occur in both DA and MB neurons, considering that AMPT ameliorated the phenotypes. The authors should confirm the possibility of DA neurons overactivity, DA hypersecretion, and tyrosine hydroxylase upregulation and Ca2+ alterations should also be confirmed in wfs1 mutant DA neurons.

*Thank you for this suggestion. The reason we focused on MB is because based on our GAL4 screen, knocking down wfs1 in Dop2R but not DA neurons lead to reduction of sleep (Fig 4A-C). Consistently, restoration of wfs1 in Dop2R neurons can rescue the short sleep phenotype of wfs1 mutants (Fig 4D). These results indicate that wfs1 activities in Dop2R neurons are both necessary and sufficient for sleep maintenance. We have now adopted a MB247GAL80 to demonstrate that wfs1 deficiency in Dop2R+ MB neurons contribute to the sleep reduction phenotype (Fig S9). In addition, we examined the expression level of tyrosine hydroxylase and DA content in these flies but observed no significant alteration (Fig S6). Taken together, we believe that wfs1 acts downstream of DA neurons in their targets, MB Dop2R neurons, to regulate sleep.*

The authors showed that knockdown of RyR rescues the phenotype, but why not also consider the involvement of IP3R?

*Thank you for this wonderful suggestion. We have now tested for genetic interaction between wfs1 and Itpr which encodes IP3R. Similar to RyR, knocking down Itpr pan-neuronally using a previously published RNAi line leads to a substantial lengthening of sleep duration in wfs1 mutants (Fig S12).*

In Table 1, isn't it an important phenotype that the power of locomotor rhythm increases with Repo drive?

*Thank you for pointing this out. Actually, the power is only increased compared to RepoGAL4/+;UASdcr2/+ control and not to UASwfs1RNAi control. This is because RepoGAL4/+;UASdcr2/+ flies display quite reduced power compared to most of the other control lines. Therefore, we believe this difference in power value is probably caused by some defects of this control line rather than wfs1 deficiency in glial cells.*

The authors should also analyze the effects of aging on Ca2+ dynamics, behavioral abnormalities, and neurodegeneration.

*Thank you for this suggestion. Since previous study has shown that wfs1 deficiency results in significant behavioral deficits and neurodegeneration in flies by 30 days of age (Sakakibara et al., 2018), we also examined the effects of wfs1 deficiency on sleep and locomotor rhythm in aged flies. We found that only one of the RNAi lines when expressed leads to significantly decreased sleep, and the extent of this reduction appears to be smaller than that of younger flies (Fig S3A-D). Aged wfs1 mutants display comparable decrease in sleep compared to that of younger flies (Fig S3E and S3F). Significant reduction of the power of locomotor rhythm can be observed in aged wfs1 RNAi and mutant flies, but the power values are relatively low even for controls which is due to aging-induced dampening of circadian rhythm (Table S2). Overall, the sleep and locomotor rhythm phenotypes associated with wfs1 deficiency do not appear to be more severe as animals age. Moreover, we found that knocking down wfs1 during development can result in reduction of sleep in adults, indicating that wfs1 is required during development for flies to maintain normal when they are adults (Fig S2). These findings imply that the behavioral defects induced by lack of wfs1 that we observe here is of neurodevelopmental origin, and thus we did not further investigate the influences of wfs1 deficiency during aging.*

Minor comments.

Please indicate directly whether Dop2R+ and Goα+ cells analyzed here are the same neurons.

*We have now analyzed the expression pattern of Dop2RGAL4 and GoαGAL4 in the MB. Dop2RGAL4 is expressed in all lobes of MB while GoαGAL4 is most prominently expressed in α and β lobes. We have also indicated this in the second paragraph of Discussion, marked in red:“Both Dop2RGAL4 and G_o_αGAL4 exhibit prominent expression in the MB, and to be more specific, in the α and β lobes of MB (Fig S7B and S7D).*”

The labeling in Fig 3C are difficult to understand, please devise new labels. It is also difficult to understand which one is significantly different from the other.

*We apologize for the difficulty that you have experienced. We have re-drawn this figure which is now Fig 4A in the revised version. We hope it is easier to understand now.*

The definitions of wake activity, the power of locomotor rhythm and % rhythm in figures and table are not clear.

*We apologize for not explaining this better in the original manuscript. We have now added more description regarding this in the second paragraph of Materials and Methods section (marked in red), which reads as: “Waking activity is calculated by dividing daily total activity during waking period by the daily duration of waking period. Chi-squared periodogram analyses of period, power, and significance values during DD was carried out using ClockLab (Actimetrics) software. For DD rhythmicity, rhythmic flies were defined as those with chi-squared power-significance ≥10. %Rhythmic is calculated as the percentage of flies of a given genotype that exhibit power-significance ≥10.”*

The graphs in Fig 4 is difficult to understand due to lack of explanation.

*We apologize for the confusion. We have now added additional labels to the figure (now Fig 5 in the revised version) to denote different temperature settings.*

The sentence "On the other hand, waking activity is not increased when these neurons are activated, which means the enhanced wakefulness is not merely due to hyper activity (Fig 4C)." should rather describe "rather decreased."

*This has been modified.*

Reviewer #2: This work by Hao et al examines the function of wolframin in the the regulation of sleep-relevant circuits, providing insight into possible mechanisms underlying sleep disorders. Given that dopamine is a conserved regulator of arousal, the findings could be generally relevant to mammals. The authors use multiple, independent genetic approaches to describe the wolframin-deficient sleep phenotype, and use a combination of drug experiments cell-type-specific rescue/KD experiments to show that wfs acts in dopaminergic neurons to regulate their activity. They go on to show that wfs influences sleep by regulating Ca homeostasis, thus providing mechanistic insight into the phenotype. While the manuscript covers a lot of ground, there are a number of areas that are confusing and require further experiments to clarify.

While the authors carry out rigorous genetic experiments to support most of their conclusions, a major conclusion of the work is that wfs negatively regulates the activity of Dop2R neurons to regulate sleep duration. These conclusions are based on Ca imaging studies whose interpretations are made difficult by two points:

-Loss of wfs1 causes opposite phenotypes using GCamP and Calexa.

-Relevance of MB lobes to sleep phenotypes not established

Given conflicting results using Ca imaging, I would have stronger faith in conclusions if authors provided additional genetic experiments to test (1) hypothesis that wfs1 limits activity of Dop2R neurons and (2) relevance of MB projecting dop2R cells to phenotype. Major revisions should include addressing:

1. Despite their pharmacologic experiments, their "cell-type-specific" GAL4 experiments for Dop2R neurons are difficult to interpret. The Dop2R-GAL4 is quite broadly expressed as is Galphao. A major conclusion of the paper is that the MB is relevant for the sleep phenotype. The authors should show this directly using intersectional approaches to label subsets of Dop2R cells projecting to MB in RNAi and/or rescue experiments. Other experiments to consider would be depletion or reduction of dop2R receptor alongside reduction of wfs1 activity, and/or silencing (e.g. Kir, shi-DN, tetanus toxin) of dop2R cells with reduced wfs1.

*Thank you for your wonderful suggestions. We have now adopted a MB247GAL80 to demonstrate that wfs1 deficiency in Dop2R+ MB neurons contribute to the sleep reduction phenotype (Fig S9). We knocked down Dop2R in wfs1 mutants and found this partially rescues the short sleep phenotype (Fig 4E), while silencing Dop2R neurons using shi^ts1^rescues the short sleep phenotype of wfs1 mutants (Fig 6D). These results strongly suggest that wfs1 deficiency in MB Dop2R neurons lead to enhanced Dop2R signaling and neural activity which result in decreased sleep.*

2. The data suggest that wfs1 is acting in an ongoing manner to regulate sleep. The authors should show that RNAi expression only the adult leads to the phenotype.

*Thank you for bringing up this important point. We used a temperature sensitive tubulin (tub)GAL80ts to knock down wfs1 specifically during the adult or developmental stage. When RNAi expression is restricted to adults, neither sleep duration nor locomotor rhythm is significantly altered (Fig S2A; Table S1). On the other hand, when RNAi is expressed exclusively during the developmental stage, sleep is significantly reduced (Fig S2B). However, when flies are raised under 29ºC, the adult locomotor rhythm is dampened even for some of the control lines (S1 Table). Therefore, we could not assess the influence of knocking down wfs1 during development on locomotor rhythm. We think this is reasonable because neurodegeneration-related symptoms start to occur within the first decade of WS1 patients, with an average age of development ranging from 11-16 years (Inoue et al., 1998; Strom et al., 1998; Rigoli et al., 2018). These findings imply that the behavioral defects induced by lack of wfs1 that we observe here is of neurodevelopmental origin.*

3. Figure 5 is very confusing and I do not fully agree with the interpretation of gcamp v calexa results. Calexa is a Ca2+ based reporter and I would expect that the results should match gcamp. For this figure to hold up, the authors need to do more to tease apart the differential results. The mixed findings in figure 6 likewise are confusing and fail to clarify - based on these results, the authors draw conclusions about genetic interactions but fail to explain a model that takes the differential results into account. This area of the manuscript requires the most work, as in current form Figures 5-6 are quite inconclusive.

*We have conducted further literature search and found that wolframin has been shown to bind to calmodulin (CaM) in rat brain, and is capable of binding with calcium/CaM complex in vitro and in transfected cells (Yurimoto et al., 2009). This may undermine the validity of using GCaMP to monitor calcium level in wfs1 deficient animals and cells, and could potentially account for the contradictory data acquired using CaLexA vs GCaMP. Therefore, we believe that CaLexA and spH are more likely to reflect the true changes of neural activity in wfs1 deficient flies, which both point to hyperexcitability. This elevated activity is consistent with our behavioral data, as activation of Dop2R/Goα+ cells reduces sleep, similar to the effects of wfs1 deficiency. Moreover, silencing Dop2R neurons rescues the short-sleep phenotype of wfs1 mutants, while over-expressing wfs1 restores the decreased sleep induced by activation of Dop2R neurons (Fig 6D and 6E). Taken together, these findings indicate that wolframin functions to suppress the excitability of Dop2R neurons, which in turn reduces wakefulness and promotes sleep.*

*We have now supplied a model figure to explain the genetic interaction results in Fig 7 (previously Fig 6), which is in Fig 7E. Hopefully this can help clarify. We have also added some description regarding this in the second to last paragraph in Discussion (marked in red): “To summarize, we believe that lack of wfs1 increases cytosolic calcium while decreasing ER calcium, leading to hyperexcitability of Dop2R neurons and thus reduced sleep (Fig 7E). Knocking down RyR or Itpr decreases cytosolic calcium and increases ER calcium, counteracting the influences of wfs1 deficiency and thus rescuing the short-sleep phenotype. On the other hand, knocking down SERCA further increases cytosolic calcium and decreases ER calcium, rendering an enhancement of the short-sleep phenotype.”*

More specific minor points on each figure follow:

Figure 1

1. All graphs in this figure should show individual data points to provide a more clear sense of data spread

*We have changed all bar graphs in this manuscript to plots with individual data points.*

Fig 3

1. Presentation of data in A is quite confusing: is AMPT treatment normalizing differences between genotypes, rescuing sleep amount, or both? Preferably should be both. Sleep values on y axis look smaller than those shown for non-drug experiments in Fig 1 and in 3B. For clarity, should include data showing effect +/- drug on genetic controls (predict sleep increase).

*We have now included data demonstrating the effect of different doses of drug treatment on the controls and experimental groups (Fig 3D and 3H). As can be seen, AMPT increases sleep in a dose-dependent manner, while this increase is more prominent in wfs1 deficient flies. Therefore, we believe AMPT is both normalizing the differences between genotypes and rescuing sleep amount.*

2. Data as shown in 3A, the rescue looks complete. Is this rescue dose-dependent? Can feeding flies L-DOPA exacerbate phenotype? Can they rescue using an independent genetic approach: KD TH using Th-GAL4 in mutant. Not satisfied with just one drug experiment.

*Thank you for the important suggestions. We have now treated the flies with different dosage of AMPT and the rescue does appear to be dose-dependent (Fig 3A and 3E). L-DOPA does not appear to substantially exacerbate the phenotype. Sleep reduction for elavGAL4;UASwfs1-RNAi flies range from 147 min to 326 min under baseline condition, depending on which control being compared to. Under L-DOPA treatment, this reduction of sleep amount range from 186 min to 274 min. Sleep reduction for wfs1 mutants is 172 min under baseline condition and 224 min under L-DOPA treatment. We also tested for genetic interaction between wfs1 and ple which encodes tyrosine hydroxylase. We knocked down ple using a previously published RNAi line and found that ple deficiency partially rescues the short-sleep phenotype and fully rescues the reduced power of locomotor rhythm in wfs1 mutants (Fig 3I; Table S3). These results support the notion that wfs1 acts via dopaminergic signaling to regulate sleep and locomotor rhythm.*

3. 3C: very hard to follow this figure, is there a way to make it easier to read?

*We apologize for the difficulty that you have experienced. We have re-drawn this figure which is now Fig 4A in the revised version. We hope it is easier to read now.*

Fig 4

1. Even though the dop2R>TrpA1 experiment has not been published to my knowledge, the dop2R null phenotype is known: sleepy flies. So the idea that Dop2R cells are wake-promoting is not new.

For what it's worth, I think the very strong wake-promoting effects seen even with gentle TrpA1 activation probably reflect the broad expression pattern of the GAL4s used.

*Yes, we agree this may be the case.*

2. There are very pronounced effects of locomotor activity in dop2R>TrpA1 and Gao>TrpA1. While I appreciate that these locomotor phenotypes are separable from sleep phenotypes (4B, 19C dark period), I would appreciate more clear description of activity phenotypes. The effects are probably light-dependent because Dop2R activity is known to be light gated.

*Thank you for this suggestion. We have now changed the description in the text regarding wake activity of wfs1 deficient flies from “not increased” to “rather decreased” (marked in red). We have also analyzed wake activity level in the light and dark phase, and found that activating Dop2R/ Goα+ cells reduces activity more prominently in the dark phase. These results are shown below.*

3. 4D-F: not sure how to match up bar graphs with sleep traces. Should make this more clear.

*We apologize for the confusion. We have now added additional labels to the figure (now Fig 5 in the revised version) to denote different temperature settings.*

4. Since wfs in Dop2R+ cells promotes sleep, what would happen to dop2R>TrpA1 phenotype in : a. M/+ b. wfs1 overepxression?

*Thank you for the wonderful suggestions. We have now conducted these experiments and found that wfs1 heterozygous mutation does not alter the sleep duration of Dop2RGAL4;UASTrpA1 flies (Fig S10). However, over-expressing wfs1 can rescue the short sleep phenotype of Dop2RGAL4;UASTrpA1 (Fig 6E), indicating that wfs1 regulates sleep by inhibiting the excitability of Dop2R neurons.*

Fig 5--Obviously very confusing w/ different Ca reporters, but also unclear about relevance of MB to wfs LOF sleep phenotypes.

1. Are these brains kept in ex vivo preps for 18h? Or are they dissecting separate brains at separate time points? Based on legend, think it's the latter. In that case, not appropriate to display data in 5B as though these were repeated measures of the same brains.

*These are separate brains dissected and imaged at separate time points. We have now changed the way we present this data (now in Fig S8B) and hopefully this will no longer be misleading.*

2. authors switch arbitrarily between reporting GFP/RFP ratios to just GFP intensity. Should be consistent across all experiments, especially for Dop2R-GAL4.

*We have now included a RFP control for all of the imaging experiments in Fig 6 and S8.*

3. 5F,G: This is confusing. Phenotype looks so dramatic. Yet how do we interpret the reduced sleep in dop2R/elavG4>wfs-IR flies?

*We believe that wfs1 deficiency leads to increased activity of Dop2R neurons, leading to enhanced wakefulness and thus reduced sleep. First of all, this is supported by the spH reporter data which indicates increased neurotransmitter release in Dop2R neurons that lack wfs1. More importantly, this is supported by our newly-added behavioral data: silencing Dop2R neurons rescues the short-sleep phenotype of wfs1 mutants, while over-expressing wfs1 restores the decreased sleep induced by activation of Dop2R neurons (Fig 6D and 6E). These findings indicate that wolframin functions to suppress the excitability of Dop2R neurons, which in turn reduces wakefulness and promotes sleep.*

Reviewer #3: In this study, authors investigated a role of WOLFRAM SYNDROME 1 (WFS1) deficiency in sleep regulation using a Drosophila model since heterozygous mutation carriers of WFS1 exhibit higher risk of psychological disorders including sleep disturbance via unknown mechanisms. They found that knocking down wfs1, a fly ortholog of WFS1, in neurons or wfs1 mutation reduced sleep. Through genetic and pharmacologic approaches, they also demonstrated that a lack of wfs1 in dopamine 2-like receptor (Dop2R) expressing neurons was responsible for observed effects through dopaminergic signaling. As for a mechanism, they proposed that wfs1 deficiency decreased cytosolic calcium via disruption of ER-mediated calcium homeostasis and increased the excitability of Dop2R expressing neurons.

This is an interesting study to add novel function of wfs1 in sleep regulation. This reviewer has several comments on the experimental design and interpretation of their results.

1. For experiments using wfs1 RNAi, it is better to used control RNAi as a control for RNAi expression.

*Thank you for this suggestion. We have now included a GFP-RNAi as a control, and expressing this RNAi using tubGAL4, elavGAL4 or repoGAL4 does not lead to significantly altered sleep or locomotor rhythm (Fig S1B; Table 1).*

2. Authors knocked down wfs1 in several different neurons including Dop2R expressing neurons and circadian clock neurons. Interestingly, however, wfs1 knockdown affected functions of Dop2R expressing neurons but not those of clock expressing neurons. Since wfs1 deficiency seemed to affect ER calcium homeostasis and neuronal excitability, it is not clear why Dop2R expressing neurons were specifically affected by wfs1 deficiency. Please clarify this point.

*Thank you for bring up this critical point. We have also been intrigued by this question but unfortunately we do not have an antibody that can label wolframin in flies. We have added some discussion in the 5^th^ paragraph of Discussion section (marked in red) regarding this issue: “It is intriguing that in our study here wfs1 deficiency appears to selectively impair the function of Dop2R/G_o_α+ neurons. It has been shown that in the rodent brain Wfs1 is enriched in layer II/III of the cerebral cortex, CA1 field of the hippocampus, central extended amygdala, striatum, and various sensory and motor nuclei in the brainstem [58-61]. Wfs1 expression starts to appear during late embryonic development in dorsal striatum and amygdala, and the expression quickly expands to other regions of the brain at birth [61]. We suspect that in flies wfs1 may be enriched in Dop2R/G_o_α+ cells during a critical developmental period, and that sufficient level of wolframin is required for their maturation and normal functioning in adults. Another possibility is that these cells are particularly susceptible to calcium dyshomeostasis induced by loss of wfs1. Indeed, this is believed to be an important cause of selective dopaminergic neuron loss in Parkinson’s Disease, as dopaminergic neurons are unique in their autonomic excitability and selective dependence on calcium channel rather than sodium channel for action potential generation [62]. We reason that Dop2R/G_o_α+ neurons may also be more sensitive to abnormal intracellular calcium concentration, making them particularly vulnerable to wfs1 deficiency.”*

3. Authors used the calcium sensor GCaMP6m to monitor calcium levels in neurons and found that basal calcium levels were decreased by wfs1 knockdown. This result is interesting; however, a relationship between low calcium levels and high neuronal excitability is a little difficult to understand. It would be important to measure changes in calcium levels upon stimulation.

*Thank you for this important suggestion. We have now examined the effect of knocking down wfs1 on dopamine-induced changes of calcium level using GCaMP6. We found that dopamine elicits a significantly smaller calcium increase in Dop2R neurons of wfs1 RNAi flies (Fig S8F). However, we have conducted further literature search and found that wolframin has been shown to bind to calmodulin (CaM) in rat brain, and is capable of binding with calcium/CaM complex in vitro and in transfected cells (Yurimoto et al., 2009). This may undermine the validity of using GCaMP to monitor calcium level in wfs1 deficient animals and cells, and could potentially account for the contradictory data acquired using CaLexA vs GCaMP. Therefore, we believe that CaLexA and spH are more likely to reflect the true changes of neural activity in wfs1 deficient flies, which both point to hyperexcitability. This elevated activity is consistent with our behavioral data, as activation of Dop2R/Goα+ cells reduces sleep, similar to the effects of wfs1 deficiency. Moreover, silencing Dop2R neurons rescues the short-sleep phenotype of wfs1 mutants, while over-expressing wfs1 restores the decreased sleep induced by activation of Dop2R neurons (Fig 6D and 6E). Taken together, these findings indicate that wolframin functions to suppress the excitability of Dop2R neurons, which in turn reduces wakefulness and promotes sleep.*

4. It would be informative to discuss the relevance of the findings to neuropathology in WS1 patients including dopaminergic system.

*Thank you for this suggestion. We have added some discussion in the 5th paragraph of Discussion section (marked in red) regarding this: “It has been shown that in the rodent brain Wfs1 is enriched in layer II/III of the cerebral cortex, CA1 field of the hippocampus, central extended amygdala, striatum, and various sensory and motor nuclei in the brainstem [58-61]. Wfs1 expression starts to appear during late embryonic development in dorsal striatum and amygdala, and the expression quickly expands to other regions of the brain at birth [61]. We suspect that in flies wfs1 may be enriched in Dop2R/Goα+ cells during a critical developmental period, and that sufficient level of wolframin is required for their maturation and normal functioning in adults. Another possibility is that these cells are particularly susceptible to calcium dyshomeostasis induced by loss of wfs1. Indeed, this is believed to be an important cause of selective dopaminergic neuron loss in Parkinson’s Disease, as dopaminergic neurons are unique in their autonomic excitability and selective dependence on calcium channel rather than sodium channel for action potential generation [62]. We reason that Dop2R/Goα+ neurons may also be more sensitive to abnormal intracellular calcium concentration, making them particularly vulnerable to wfs1 deficiency. The pathogenic mechanism underlying the neurodegeneration of WS1 is quite complex, possibly involving brain-wide neurodegenerative processes and neurodevelopmental disregulations [4]. Our findings here provide some evidence supporting a role for altered dopaminergic system during development. Obviously, much more needs to be done to test these hypotheses.”*

Have all data underlying the figures and results presented in the manuscript been provided?

Large-scale datasets should be made available via a public repository as described in the PLOS Genetics data availability policy, and numerical data that underlies graphs or summary statistics should be provided in spreadsheet form as supporting information.

Reviewer #1: No: The origin of all the fly strains used is not stated.

*This has been added.*

Reviewer #2: Yes

Reviewer #3: Yes
