# Supplementary material for: Wolfram syndrome 1 regulates sleep in dopamine receptor neurons by modulating calcium homeostasis
Source: PLoS Genet. 2023 Jul 3;19(7):e1010827. doi: 10.1371/journal.pgen.1010827 (PMC10348591; doi:10.1371/journal.pgen.1010827)
Supplement: Attachment — Submitted filename: response to reviewers 6-4-23.docx [file pgen.1010827.s021.docx]

***We thank the reviewers for their thoughtful comments and suggestions which have made the manuscript much stronger. We have addressed these comments and suggestions as described below. The original reviews are listed point-by-point. Our responses are in blue italic font.* *Edits made in the text of the manuscript are marked in red.***

Reviewer #1: This reviewer is approximately satisfied with the authors' response. However, the interpretation of the GCaMP results remains a concern and the results do not support the hypothesis in Fig 7E.

The authors point out the possibility that wfs1 binds to the CaM region of GCaMP and affects GCaMP activity. However, this would need to be demonstrated experimentally. For example, it would be possible to overexpress wfs1 in unrelated tissues and see its effect on GCaMP activity. Or Ca2+ activity of neurons should be measured in modalities other than GCaMP.

*We agree that the GCaMP results do not support the hypothesis in Fig 7E. This hypothesis is mainly based on the genetic interaction data in Fig 7. We have now indicated this in the second to last paragraph of Discussion section which is marked in red (“Based on the results of these genetic interactions, we propose that lack of wfs1 increases cytosolic calcium while decreasing ER calcium, leading to…”). We fully agree that more experiments (perhaps much more), including the ones that you suggested, are required to elucidate this issue. However, these may take quite a bit of time and efforts that likely extend beyond the scope of this current study. We would certainly like to further investigate this matter in our future work, and for this revision, as suggested by the editor and the other reviewer, we have scaled back the conclusions drawn from these and other imaging data. We have changed the claim of “hyperexcitability” being the underlying mechanism to “altered excitability” to acknowledge there is a possibility of lowered excitability suggested by the GCaMP data (marked in red in the Abstract, Introduction, Results, Discussion and figure legend). When discussing about the GCaMP results, we have also indicated that the results may reflect decreased excitability (the fourth paragraph of Discussion, marked in red). Regarding the hypothesis in Fig 7E, we have added more discussion on these caveats (end of the second to last paragraph of Discussion section, marked in red) which reads as: “We do acknowledge that the hypothesis proposed in Fig 7F is not supported by our GCaMP data, which indicates decreased cytosolic calcium level in Dop2R neurons of wfs1 deficient flies. We suspect that since the sleep phenotype associated with lack of wfs1 is of developmental origin, it is possible there is an initial increase of cytosolic calcium during critical developmental period in wfs1 deficient flies and this influences the function of Dop2R neurons in adults. Clearly, further characterizations need to be done to fully elucidate this issue, and preferably another calcium indicator independent of the GCaMP system should be employed.” We hope this would be satisfactory to you for now and we truly appreciate your suggestions to help improve our manuscript.*

Reviewer #2: The authors have done a tremendous amount of work in this revision, and attempted to address concerns of all the reviewers. While there are still some remaining issues with regard to interpretation of the calcium measures, I think this work is a nice addition to the literature. The new genetic expts (dop2R KD, dop2R neuron silencing) improve the rigor of the conclusions from the behavioral data. The GAL80ts result likewise adds a nice conclusion, showing that the sleep phenotype is of developmental origin. There are two issues that remain and should be addressed with changes to text.

1. The MB GAL80 experiments are not very convincing (Fig S9). At a minimum, the authors should say that MB GAL80 attenuates the phenotype (does not abolish it) and leave open the possibility that non MB regions contribute. One could argue that the authors should add the proper positive controls (full sleep phenotype) to this experiment to directly compare, as it is possible that in these particular experimental runs that the MB GAL80 does not impact the phenotype at all, meaning it is of non MB origin. Since this is unlikely, I would be satisfied with just a softening of the language - there is indeed still a sleep loss phenotype with RNAi in the setting of MB GAL80, perhaps just not as big.

*Thank you for this suggestion. We have now changed “abolishes” to “attenuates”.*

2. I am still not satisfied with their interpretation of the Ca imaging data. The conclusions are not backed up by the experiments. I think the most telling result, which is somewhat buried in a supplemental figure, is that wfs is required for Dop2R neuronal activity in response to exogenous DA. This stands in direct contrast to idea of hyperexcitability being the underlying mechanism. But the authors call into question whether the results are reliable because of a potential interaction with wfs and GCaMP. While this is theoretically possible, I do not think it is sufficient grounds to disregard the data in favor of results that better fit their hypothesis. At this stage, however, I do not think it is reasonable to request more experiments. Instead, I favor really drawing back the conclusions and discussion as it pertains to hyperexcitability and the calcium results in general. The authors did a ton of work and this should be shown! But I think they can keep the discussion on issues with gcamp v calexa and have the idea of hyperexcitability as more speculative in the discussion since the results do not fully back this up.

*Thank you for this suggestion to improve the rigor of our manuscript. We have now changed the claim of “hyperexcitability” being the underlying mechanism to “altered excitability” to acknowledge there is a possibility of lowered excitability suggested by the GCaMP data (marked in red in the Abstract, Introduction, Results, Discussion and figure lengend). When discussing about the GCaMP results, we have also indicated that the results may reflect decreased excitability (the fourth paragraph of Discussion, marked in red). Regarding the hypothesis in Fig 7E, we have added more discussion on these caveats (end of the second to last paragraph of Discussion section, marked in red) which reads as: “We do acknowledge that the hypothesis proposed in Fig 7F is not supported by our GCaMP data, which indicates decreased cytosolic calcium level in Dop2R neurons of wfs1 deficient flies. We suspect that since the sleep phenotype associated with lack of wfs1 is of developmental origin, it is possible there is an initial increase of cytosolic calcium during critical developmental period in wfs1 deficient flies and this influences the function of Dop2R neurons in adults. Clearly, further characterizations need to be done to fully elucidate this issue, and preferably another calcium indicator independent of the GCaMP system should be employed.”*
